# Prognostic values of long noncoding RNA in bone metastasis of prostate cancer: A systematic review and meta-analysis

**DOI:** 10.3389/fonc.2023.1085464

**Published:** 2023-02-20

**Authors:** Silu Song, Yanli Zhu, Xue Zhang, Siyu Chen, Shuang Liu

**Affiliations:** Key Laboratory of Microecology-Immune Regulatory Network and Related Diseases, Department of Basic Medical, Jiamusi University, Jiamusi, China

**Keywords:** lncRNA, prostate cancer, bone metastasis, prognosis, meta-analysis

## Abstract

**Introduction:**

Recent studies have shown that long non-coding RNAs are closely related to the occurrence and development of prostate cancer bone metastasis, and can be used as biomarkers to predict the prognosis of patients. Therefore, this study aimed to systematically evaluate the relationship between the expression levels of long non-coding RNAs and the prognosis of patients.

**Methods:**

The studies of lncRNA in prostate cancer bone metastasis from Pubmed, Cochrane library, Embase, Ebsco, Web of science, Scopus, Ovid databases were analyzed, and Stata 15 was used for meta-analysis. Associations between lncRNA expression and patients’ overall survival (OS) and bone metastasis-free survival (BMFS) were assessed by correlation analysis with pooled hazard ratios (HR) and 95% confidence intervals (CI). Furthermore, the results were validated using GEPIA2 and UALCAN, online database based on TCGA. Subsequently, the molecular mechanisms of the included lncRNAs were predicted based on the LncACTdb 3.0 database and the lnCAR database. Finally, we used clinical samples to validate lncRNAs that were significantly different in both databases.

**Results:**

A total of 5 published studies involving 474 patients were included in this meta-analysis. The results showed that lncRNA overexpression was significantly associated with lower OS (HR = 2.55, 95% CI: 1.69 - 3.99, *p* < 0.05) and lower BMFS (OR = 3.16, 95% CI: 1.90 - 5.27, *p* < 0.05) in patients with prostate cancer bone metastasis. Based on validation from the GEPIA2 and UALCAN online databases, SNHG3 and NEAT1 were significantly up-regulated in prostate cancer. Further functional prediction showed that the lncRNAs included in the study were involved in regulating the occurrence and development of prostate cancer through the ceRNA axis. The result of clinical samples showed that SNHG3 and NEAT1 were expressed in prostate cancer bone metastasis at higher levels than in primary tumors.

**Conclusions:**

LncRNA can be used as a novel predictive biomarker for predicting poor prognosis in patients with prostate cancer bone metastasis, which is worthy of clinical validation.

## Introduction

Prostate cancer is the most common cancer of the male reproductive system. Its effects increase with age (1), and threatens men’s health. Prostate cancer has the highest death rate among male cancer deaths, mainly due to metastatic spread of prostate cancer cells to different organs, including bone (2). After bone metastasis in patients with prostate cancer, patients typically have a reduced quality of life, worse prognoses, and increased frequency of pain and bone-related events (3).

Prostate cancer has a strong tendency to metastasize to bone, and studies have found that 65% to 75% of prostate cancer patients develop bone metastasis (4).The occurrence of bone metastasis obviously affects the choice of treatment for prostate cancer (5).At present, the main methods for the diagnosis and prognosis of prostate cancer bone metastasis are imaging methods (68Ga-PSMA-PET/CT (6), 18F-NaF-PET/CT (7), Whole-body magnetic resonance imaging (WB-MRI) (8), etc.), PSA level detection (9), genomic analysis (lncRNA, miRNA, mRNA research), etc. In recent years, more and more studies of prostate cancer bone metastasis have focused on lncRNAs because of their important roles in human disease. In recent years, more and more studies of prostate cancer bone metastasis have focused on lncRNAs because of their important roles in human disease.

Long non-coding RNA (lncRNA) is one of the non-coding RNAs, which refers to non-coding RNA longer than 200 nucleotides (10). Recently, lncRNAs mainly transcribed from intergenomes have become a research focus of cancer transcriptome (11). Many studies have shown that lncRNAs are important regulators of gene expression, affecting the expression of protein-coding mRNA through co-expression or competing endogenous RNA (ceRNA) models, acting nearby (cis) or distant (trans) protein-coding genes, thereby affecting tumor progression (12). It plays an important role in epigenetic regulation, cell differentiation regulation, and cell cycle regulation. In addition, it participates in various biological processes through different mechanisms, and plays an important role in tumor proliferation, invasion, migration, and apoptosis. A variety of specific lncRNAs play important roles in the development, metastasis and prognosis of prostate cancer bone metastasis. Misawa et al. (13) found that HOXA11-AS and HOXB13 regulated CCL2/CCR2 cytokines and integrin signaling pathways through autocrine and paracrine ways to promote prostate cancer bone metastasis, in which HOXB13 is an upstream regulator of lncRNA HOXA11-AS. Jiang et al. (14) found that castration-resistant prostate cancer cell-derived exosomal HOXD-AS1 acted as a ceRNA that binds to miR-361-5p and acted on the target FOXM1 to form HOXD-AS1/miR-361-5p/FOXM1 axis, which in turn promoted bone metastasis of prostate cancer. Sebastian et al. (15) found that osteoblast Sost as a major regulator of gene expression in prostate cancer cells, and identified MALAT1 as a target gene regulated by Sost in prostate cancer cells. Their further research showed that the regulation of Sost/Wnt/MALAT1 pathway might be a new way of therapeutic intervention for the treatment of prostate cancer bone metastasis.

It can be seen that lncRNA may be a potential and novel prognostic diagnostic marker for prostate cancer patients with bone metastasis. Based on the differences of studies, there is currently no unified opinion on lncRNAs as biomarkers. Evidence-based medicine is a common statistical method used to compare and synthesize the results of studies on the same scientific question, which conclusions are meaningful or not depends on the quality of included studies, and is often used in quantitative combined analyses in systematic reviews. Compared with a single study, by integrating all relevant studies, the effect of medical and health care can be more accurately estimated, and it is conducive to exploring the consistency of research evidence and the differences between studies. When the results of multiple studies are inconsistent or have no statistical significance, Meta-analysis can be used to obtain statistical analysis results that are close to the real situation. Therefore, we integrated relevant studies into the meta analysis to determine the prognostic value of lncRNA expression in prostate cancer bone metastasis, and to explore the potential of lncRNA as a novel biomarker and therapeutic target for prostate cancer bone metastasis.

## Methods

### Literature search strategy

The English electronic databases (Pubmed, Cochrane library, Embase, Ebsco, Web of science, Scopus, and Ovid) were comprehensively searched and studies on the correlation between lncRNA expression and prognosis of patients with prostate cancer bone metastasis published abroad before February 2022 were collected. The search terms include: (“RNA, Long Noncoding” OR “lncRNA” OR “Long Non Coding RNA” OR “Long Non Protein Coding RNA” OR “Long Noncoding RNA” OR “Long Untranslated RNA” OR “LincRNA” OR “LINC”) AND (“Prostatic Neoplasms” OR “Prostate Neoplasm” OR “Prostate Cancer” OR “Prostate Tumor”) AND (“Bone metastases” OR “Bone metastasis” OR “Skeletal metastasis” OR “Osseous metastasis”). References of relevant studies were also searched to avoid omitting any potentially eligible studies.

### Inclusion and exclusion criteria

Inclusion criteria: (1) The survival information of patients can be reflected by valuable data, such as overall survival (OS) rate, bone metastasis-free survival (BMFS) rate; (2) Detect the expression level of lncRNA in pathological specimens of patients by specific experimental methods such as quantitative real time polymerase chain reaction (qRT-PCR), *in situ* hybridization (ISH), or other experimental methods; (3) Hazard ratios (HR) and 95% confidence interval (CI) can be obtained directly from the article or indirectly according to the Kaplan-Meier curve; (4) The patient has not received anti-tumor therapy such as radiotherapy, chemotherapy, molecular targeted drug therapy before surgery.

Exclusion criteria: (1) Duplicate articles; (2) Conference abstracts, case reports, reviews, meta-analyses, letters, patents, expert opinions, unpublished studies; (3) Article data are from the TGCA database; (4) Non-human research studies; (5) The studies in the article are not related to LncRNA, prostate cancer, and bone metastasis; (6) The study data in the article are incomplete or lack high-quality clinical data.

### Articles screening and data extraction

To ensure the accuracy of data extraction, two authors extracted data according to the inclusion and exclusion criteria, and a third author assisted in making the final decision on the disputed information. The extracted data included: first author, publication year, country, study duration, sample size, sample type, lncRNA type and expression in patients with prostate cancer bone metastasis, detection methods, prognostic indicators, HR and 95% CI, follow-up. There are two ways to obtain HR and 95% CI: one is obtaining data directly provided in the included article; or, if the included article provides Kaplan-Meier curve, the relevant data is obtained indirectly using Engauge Digitizer 11.1 software (16).

### Articles quality evaluation

The Newcastle-Ottawa Scale (NOS) was used to evaluate the quality of the included articles (17). The evaluation included 3 columns (selection, comparability, exposure) and 8 items. The full score of this evaluation is 9 points, with 0-5 as low-quality article, 6-9 as high-quality article. High-quality article can be used for final meta-analysis. The article quality evaluation process was carried out by two researchers independently. When there was a disagreement in the quality evaluation, the two researchers negotiated and discussed, or a third researcher resolved the disagreement.

### Bioinformatics database validation

Gene Expression Profile Interaction Analysis 2 (GEPIA2) (18) (http://gepia2.cancer-pku.cn/) is a website for online bioinformatics analysis based on RNA sequencing expression data from The Cancer Genome Atlas (TCGA) and GTEx projects, which can realize fast and custom functions. This online tool can be used to further verify the expression levels of the included lncRNA in the meta-analysis in prostate cancer tissue. One-way ANOVA was used to analysis the gene differential expression and *p*<0.01 is the critical value. UALCAN (19, 20) (http://ualcan.path.uab.edu/index.html) is a comprehensive, user-friendly, interactive web resource for analyzing cancer OMICS data for online analysis and mining, primarily based on relevant cancer data from the TCGA database.

### Functional prediction of lncRNA

We performed predictions of associated miRNA and mRNA for the lncRNA included in the studies. LncACTdb 3.0 (21) (http://bio-bigdata.hrbmu.edu.cn/LncACTdb/index.html) is a comprehensive database based on the analysis of TCGA and GEO datasets, containing experimentally supported interactions, know as ceRNA networks, which we used to predict the target genes of the lncRNAs included in the study. LnCAR (22) (https://lncar.renlab.org/) is developed based on the GEO database platform to explore lncRNA obtained by reannotating gene expression microarray data, and this database was used to predict the miRNA of lncRNA included in the study. Finally, a network of lncRNA-mRNA and lncRNA-miRNA interactions was constructed using Cytoscape software (23) (version 3.9.1).

### Clinical samples and quantitative real-time polymerase chain reaction

Serum samples were collected from 17 primary tumors and 17 bone metastasis patients at the First Affiliated Hospital of Jiamusi University. None of the patients had received any treatment prior to serum collection. All Serum samples were frozen at -80 °C. Each participant written consent and the ethics committee of the First Affiliated Hospital of Jiamusi University agreed this study.

Total RNA in serum was extracted using Trizol (Beyotime, China) reagent, cDNAs were synthesized with Beyotime, qRT-PCR was conducted using PowerUpTM SYBRTM Green Master Mix (Thermo Fisher Scientific) on ABI 7300 (Thermo Fisher Scientific) system. Actin was selected as internal reference. Raw data of SNHG3 and NEAT1 were normalized to Actin. The relative expressions were calculated by comparative Ct (2^−ΔΔCt^) method. The primers (forward and reverse) were listed in **Table 1**.

**Table 1:** Forward and reverse primers sequences.

Name	Forward primer	Reverse primer
β-actin	CCTGGCACCCAGCACAAT	GGGCCGGACTCGTCATAC
NEAT1	CCTCACCTATCCCACCCTACTACAC	CTCTCCCTCCCTCTGCCTTCAC
SNHG3	CAGCCGTTAAGCCATTTGGAACTTG	CAACCCTGACCTCAACACCTTGG

### Statistical analysis

Meta-analysis was performed with Stata 15 software. The combined HR value and 95%CI were used to count the data effect quantity. If HR>1, 95%CI did not include 1 and *p*<0.05, it indicated that the prognosis was poor, and the difference was statistically significant. For the problem of heterogeneity, Q-test and *I^2^* test were used to evaluate the statistical heterogeneity among the studies. When *I^2^* <50% and *p*>0.05 of Q-test, it indicates that the heterogeneity across the studies is small, and the fixed effect model is used; when *I^2^* ≥50% and *p ≤* 0.05 of Q test, it indicates that there is significant heterogeneity among various studies. If there is heterogeneity in the results, the factors causing heterogeneity can be found through subgroup analysis. Sensitivity analysis was used to evaluate the stability of the combined results. Begg and Egger methods were used to evaluate whether there was publication bias in meta analysis. *p*<0.05 showed that the difference was statistically significant.

## Results

### Article search results

**Figure 1:**
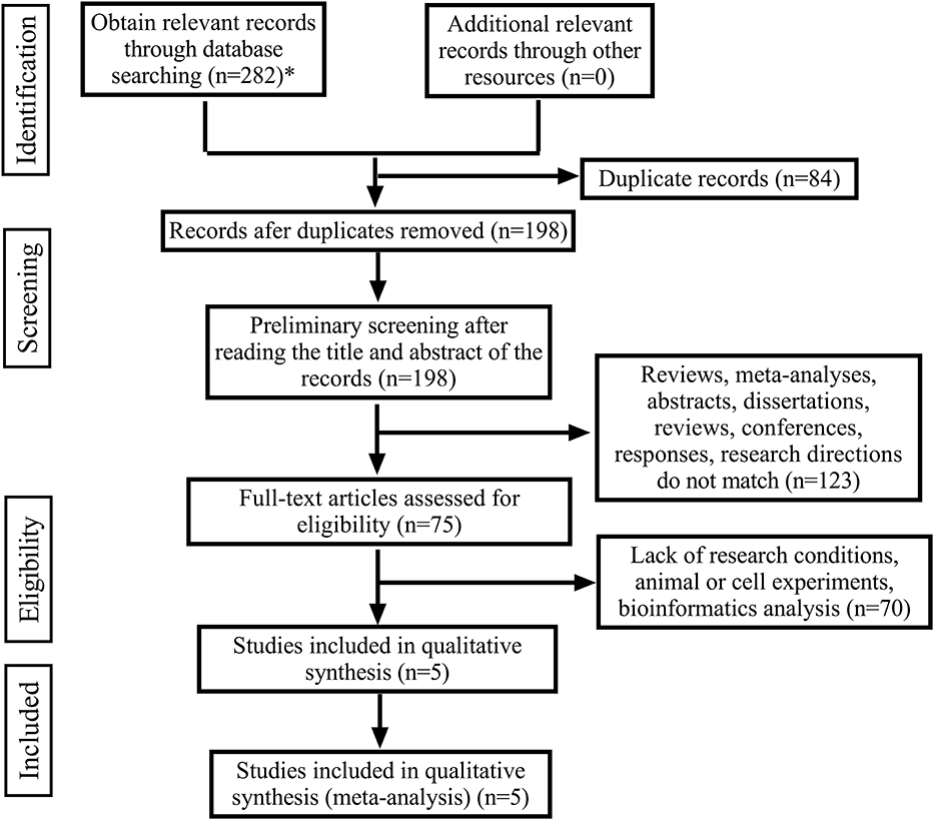
The flow diagram of the publication search, selection and exclusion process. (*The databases searched and the number of documents detected are as follows: Pubmed (n=21), Cochrane library (n=0), Scopus (n=131), Ovid (n=9), Embase (n=17), Ebsco (n=40), Web of Science (n=64)).

From English databases (Pubmed, Cochrane library, Scopus, Ovid, Embase, Ebsco, Web of Science) according to the established search strategy, a preliminary search was carried out, and a total of 282 articles were retrieved. Using Endnotes X9 software, 84 duplicate articles were screened out. After reading the titles and abstracts of the articles, 123 articles were excluded. After further reading the full text, 70 articles were excluded due to lack of qualified prognostic information. Finally, 5 articles met the selection criteria and were included in this meta-analysis, with a total of 474 patients (**Figure 1**).

### Basic characteristics and quality scores of included articles

5 articles included in the meta-analysis were published from 2020 to 2022. In these 5 studies on the relationship between lncRNA expression levels and patients with prostate cancer bone metastasis, a total of 474 patients were included. All included studies were all from China. The expression of lncRNA in the included articles was detected by qRT-PCR. NOS was used to evaluate the quality of all included articles, and quality evaluation results were 6-8 points, indicating that the quality of the 5 included articles was high. The basic characteristics and quality evaluation of eligible studies were shown in **Table 2**.

**Table 2:** Main characteristics of all eligible studies included in the meta-analysis.

First author	year	country	Recruitiment time	Sample size(n)	Sample type	LncRNA type	Detection method	Outcome	Source of HR	Follow-up months	NOS scores
Chuandong Lang (24)	2021	China	NR	163	Tissue	PCAT6	qRT-PCR	OS/BMFS	①	60	6
Xinhua Xi (25)	2022	China	2010-2017	60	Tissue	SNHG3	qRT-PCR	OS/BMFS	①	60	8
Simeng Wen (26)	2020	China	1995-2018	50	Tissue	NEAT1	qRT-PCR	OS	②	20	7
Qier Xia (27)	2020	China	2011-2019	127	Tissue	SNHG7	qRT-PCR	OS	①	60	8
Juan Chen (28)	2021	China	2011-2015	74	Tissue	NORAD	qRT-PCR	OS	②	60	7

NR, not reported; OS, overall survival; BMFS, bone metastasis-free survival; HR, hazard ratio; ①: directly provided; ②: survival curve.

### Relationship between lncRNA expression level and OS in prostate cancer patients with bone metastasis

**Figure 2:**
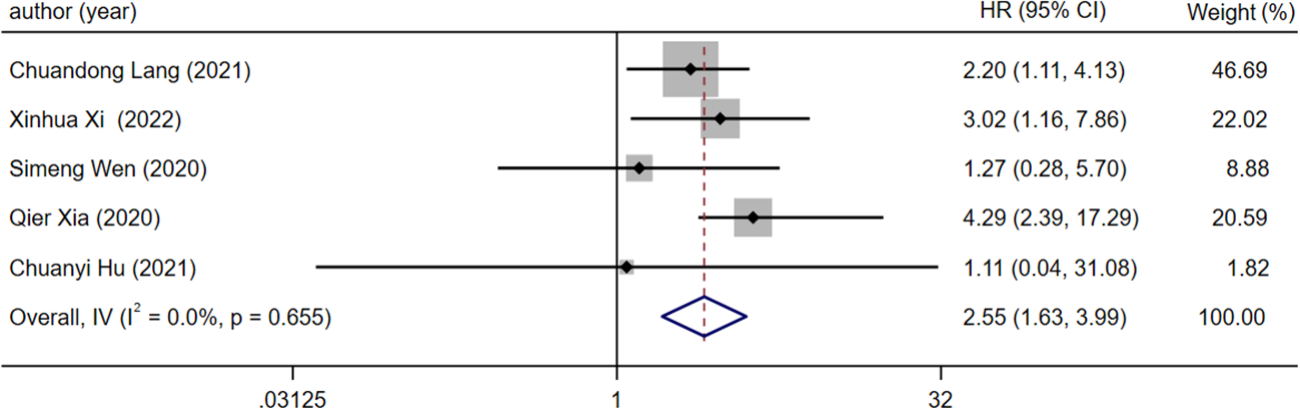
Forest plot of meta-analysis of the relationship between lncRNA expression and OS in prostate cancer patients with bone metastasis.

Five articles were all reported overall survival (OS) and different expression levels of lncRNA. After the heterogeneity test (*I^2 =^* 0.00%, the Q test *p*=0.655), the fixed effect model was used for analysis. The results showed that the combined effect size HR=2.55, 95%CI was 1.63-3.99, and there was a significant correlation between the expression level of LncRNA and OS of patients (z=4.080, *p*<0.05), which means that compared with patients with low lncRNA expression, patients with high lncRNA expression have lower OS (**Figure 2**).

### Relationship between lncRNA expression level and BMFS in prostate cancer patients with bone metastasis

**Figure 3:**
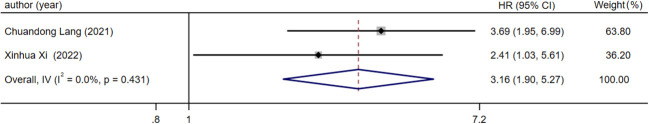
Forest plot of meta-analysis of the relationship between lncRNA expression and BMFS in prostate cancer patients with bone metastasis.

There were two articles reported BMFS with different expression levels of lncRNA. After the heterogeneity test (*I^2 =^* 0.00%, Q test *p*=0.431), there is no heterogeneity in the research results, so the fixed effect model is used for analysis. The results showed that the combined effect size HR=3.16, 95%CI was 1.90-5.27, and there was a significant correlation between the expression level of lncRNA in prostate cancer bone metastasis and the BMFS of patients (z=4.426, *p*<0.05), meaning that compared with patients with low lncRNA expression, patients with high lncRNA expression had lower BMFS (**Figure 3**).

### Sensitivity analysis and publication bias

**Figure 4:**
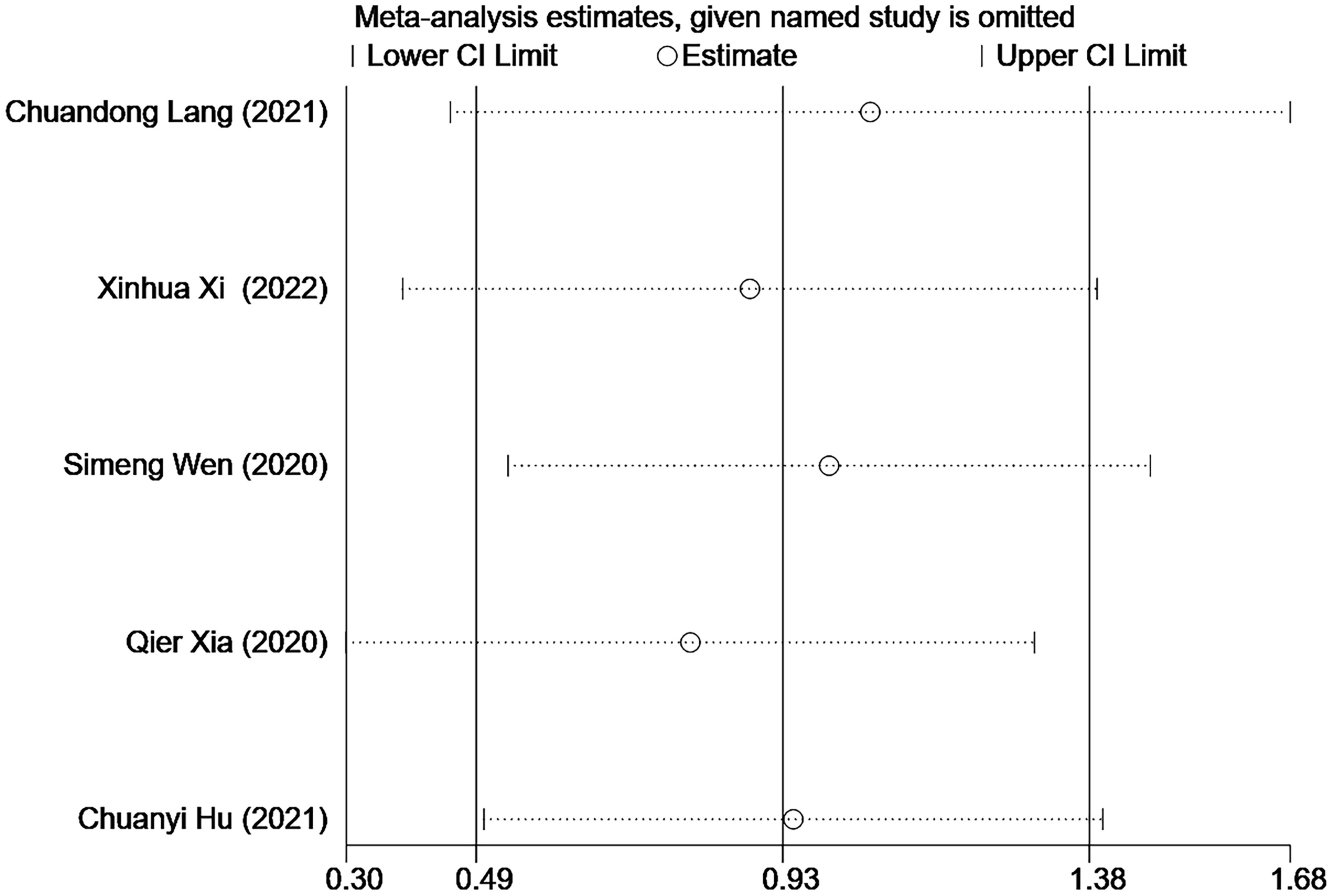
Sensitivity Analysis.

Due to the lack of data on BMFS, this study only evaluated the results of OS. The results showed that two articles (24, 27) had a greater impact on the study results, and the results of other included studies were stable (**Figure 4**). Due to the relatively limited number of included articles, publication bias could not be satisfied, so publication bias was not performed.

### Validate results in GEPIA2 database and UALCAN database

**Figure 5:**
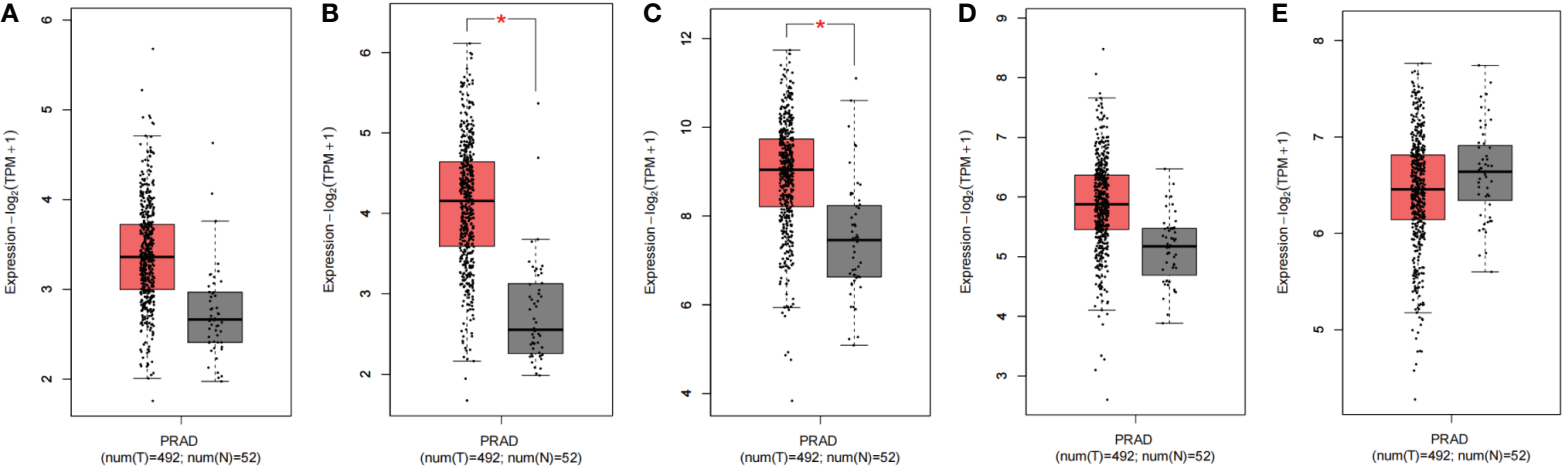
Expression levels of five lncRNA in prostate cancer tissues and normal tissues. **(A)** PCAT6; **(B)** SNHG3; **(C)** NEAT1; **(D)** SNHG7; **(E)** NORAD. “∗”means |Log2Fold Change(FC)|>1 and *p*<0.01. The red box plots represent genes expressed in cancer tissues, and the grey box plots represent genes expressed in normal tissues.

**Figure 6:**
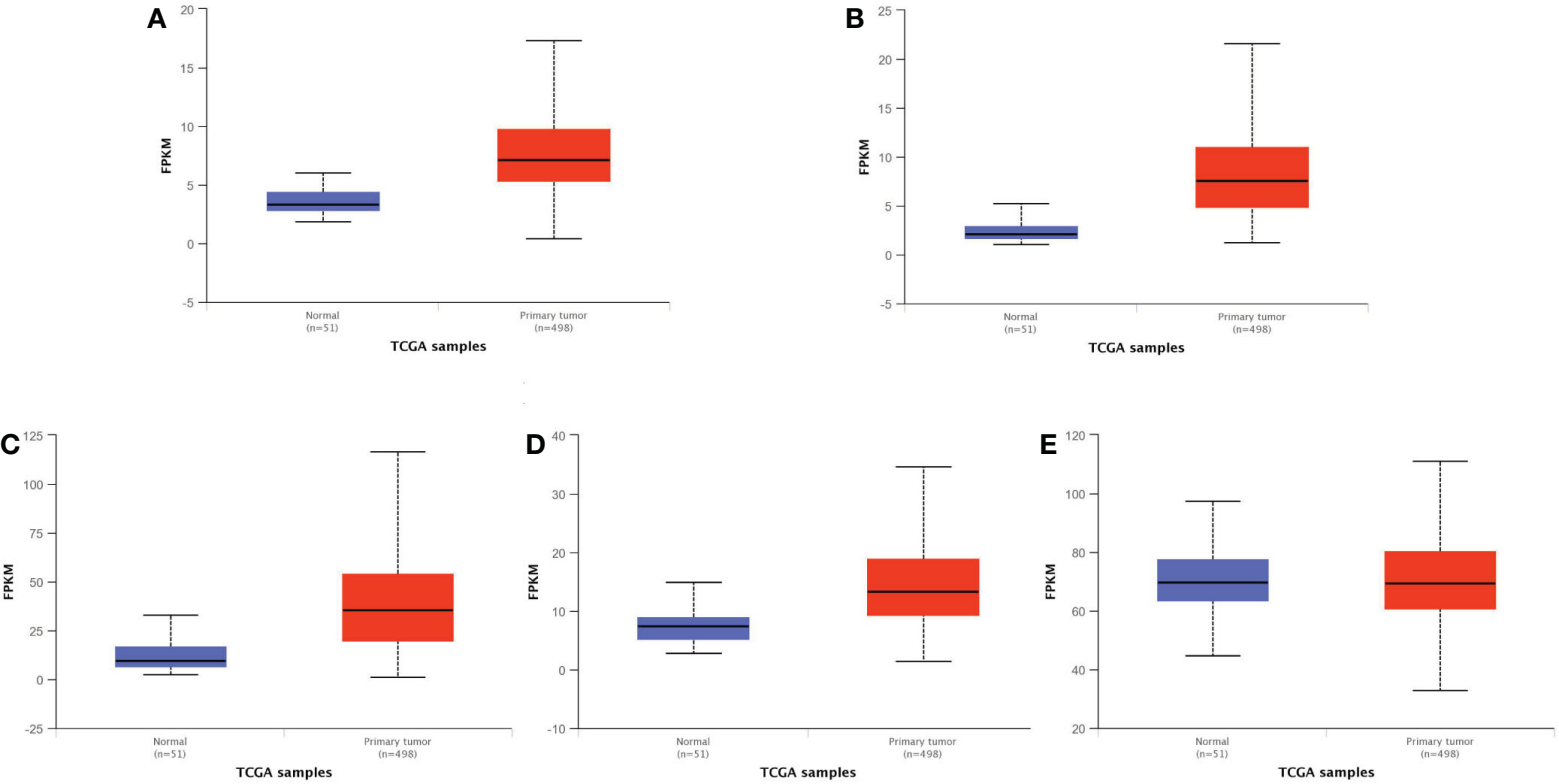
Expression levels of five lncRNA in normal and primary tumor. **(A)** PCAT6; **(B)** SNHG3; **(C)** NEAT1; **(D)** SNHG7; **(E)** NORAD. Difference is extremely significant in **(A–D)**, *p* < 0.001. There is no difference between them in **(E)** The bule box plots represent genes expressed in normal, and the red box plots represent genes expressed in primary tumor.

To further validate our results, we used GEPIA2 to evaluate the expression levels of SNHG3, SNHG7, NEAT1, PCAT6, and NORAD in prostate cancer, and the results showed that SNHG3, SNHG7, NEAT1, PCAT6, and NORAD were up-regulated in prostate cancer, but based on the database results, only the differential expressions of SNHG3 and NEAT1 in prostate cancer were statistically significant (|Log_2_FC| Cut-off: 1, *p*-value Cut-off: 0.01) (**Figure 5**). We also evaluated the expression levels of SNHG3, SNHG7, NEAT1, PCAT6 and NORAD in prostate cancer using ULCAN, and the results showed that the differential expression of SNHG3, SNHG7, NEAT1 and PCAT6 in prostate cancer was statistically significant (**Figure 6**). The reason for the differential results between the two databases, we guess that it may be strongly related to the use of different calculation criteria, but it can be seen that SNHG3 and NEAT1 are statistically significant by the two databases.

### Prediction results of the functions of lncRNA in included studies

**Figure 7:**
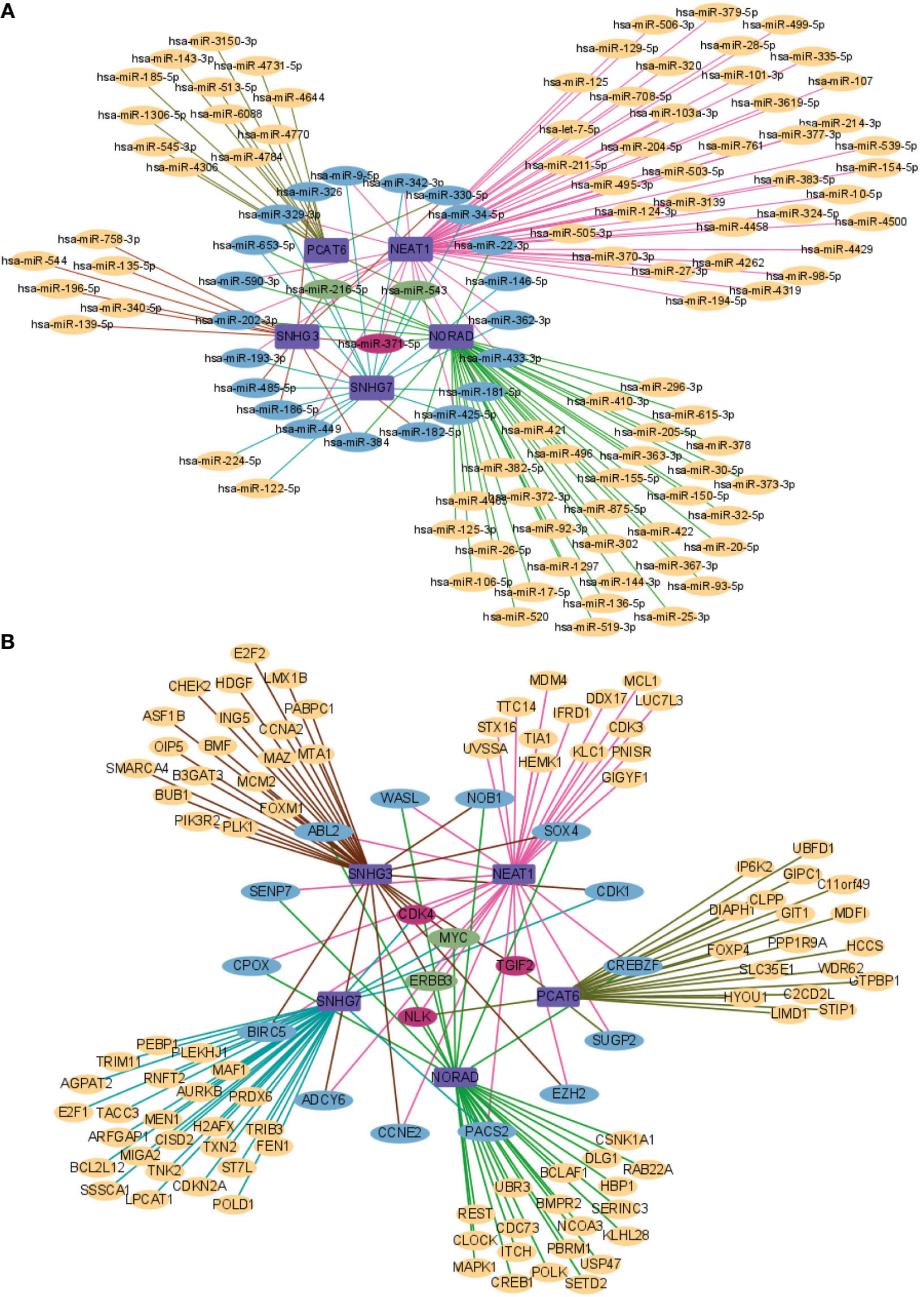
Network analysis of lncRNA and targeted genes. **(A)** lncRNA and miRNA network analysis; **(B)** lncRNA and mRNA network analysis.

In order to further understand the molecular mechanism of lncRNA in included studies regulating bone metastasis of prostate cancer. We used the lnCAR database to predict miRNA of the lncRNA included in the study, since each lncRNA has a large number of analysis ID, we selected the optimal one according to |Log_2_FC|>1 and *p*<0.05 to make the lncRNA-miRNA network diagram, among which miR-9-5p, miR-326, miR-329-3p, miR-653-5p, miR-590-3p, miR-202-3p, miR-193-3p, miR-485-5p, miR-186-5p, miR-449, miR-384, miR-182-5p, miR-425-5p, miR-181-5p, miR-433-3p, miR-362-3p, miR-146-5p, miR-22-3p, miR-34-5p, miR-330-5p, and miR-342-3p have two lncRNA co-targeting; miR-371-5p has three lncRNA co-targeting; miR-216-5p and miR-543 are co-targeted by four lncRNA (**Figure 7A**). Then, we used the LncATCdb 3.0 database to predict mRNA of the included lncRNA. There are too many mRNA targeted by each lncRNA. According to the sample size, we selected the ones with a large number of samples and those targeted by multiple lncRNAs to make the lncRNA-miRNA network diagram. Among them, WASL, ABL2, SENP7, CPOX, BIRC5, ADCY6, CCNE2, PACS2, EZH2, SUGP2, CREBZF, CDK1, SOX4, NOB1 each has two lncRNA co-targeting; CDK4, TGIF2, NLK each has three lncRNA co-targeting; MYC and ERBB3 are co-targeted by four lncRNAs (**Figure 7B**). Therefore, more studies are needed to explore the underlying molecular mechanisms of the interaction of lncRNA with miRNA and mRNA to identify new biomarkers for prostate cancer bone metastasis.

### Expression of SNHG3 and NEAT1 in primary tumors and bone metastasis of prostate cancer

**Figure 8:**
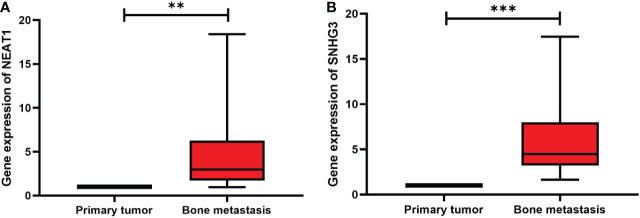
Expression levels of two lncRNAs in primary tumors and bone metastasis of prostate cancer. **(A)** NEAT1; **(B)** SNHG3. **p < 0.01, ***p < 0.001.

To verify the expression of lncRNA SNHG3 and NEAT1 in bone metastasis of prostate cancer, we performed qRT-PCR assays in clinical serum samples. Both lncRNAs were significantly increased in the serum of patients with bone metastasis compared with the primary tumor (**Figures 8A, B**).

## Discussion

With the rise of RNA sequencing, lncRNAs have been rapidly developed, covering much more sites in the human genome than protein-coding genes. LncRNAs regulate protein-coding genes at multiple levels and play a key role in genomic imprinting, cell differentiation, and cancer progression (29). Studies have reported that lncRNA are abnormally differentially expressed in cancers, which pathogenesis has not yet been clarified. Generally speaking, lncRNA dysregulation promotes cancer cell proliferation, migration, invasion and other malignant biological behaviors to promote the occurrence of cancer. LncRNAs have the characteristics of high stability and strong tissue specificity, and can be used as potential targets and biomarkers for tumor therapy and used for clinical diagnosis and prognosis evaluation.

In this meta-analysis, the results showed that high lncRNA expression was significantly associated with shorter OS and BMFS in prostate cancer bone metastasis. Therefore, the condition and prognosis of patients can be comprehensively analyzed and evaluated by detecting the expression of lncRNA, providing a valuable reference for targeted treatment of prostate cancer patients with bone metastasis. As far as we consult many articles, this study is the first meta-analysis to focus on the prognostic value of lncRNA in prostate cancer bone metastasis.

Dozens of studies found that lncRNAs influence the development of prostate cancer bone metastasis through different mechanisms. Based on bioinformatics analysis, Chen et al. (30) predicted that lncRNAs (HCG18 and MCM3AP-AS1) were closely associated with bone metastasis and poor prognosis in prostate cancer, and further analyzed that HCG18 may be involved in prostate cancer bone metastasis by affecting the expression of KNTC1 and hsa-miR-127-3p-CDKN3. Zhang et al. (31) found that HOTAIR could form ceRNA through sponging miR-520b *in vitro*, up-regulating the expression of target gene FGFR1, and eventually promoting bone metastasis of prostate cancer. Lang et al. (32) found that PCAT7 up-regulated the expression of TGFBR1 by sponging miR-324-5p, thereby activating TGF-β signaling and promoting the occurrence of prostate cancer bone metastasis. Their further research found that the SMAD3/SP1 transcription complex promoted the transcription of PCAT7 and formed a structurally active loop between PCAT7 and TGF-β signaling, which promoted bone metastasis in prostate cancer as well.

The five included lncRNAs have also been extensively studied by many researchers in prostate cancer, and we elaborate on their biological mechanisms and functions in prostate cancer. Chen et al. (33) found that lncRNA NORAD forms ceRNA with miR-495-3p *in vitro*, which acts on mRNA TRIP13 and constitutes the NORAD/miR-495-3p/TRIP13 axis to regulate the biological behavior of LNCaP apoptosis, proliferation, migration, and invasion in prostate cancer cells, providing a new insight into the treatment of prostate cancer. Zhang et al. (34) found that NORAD targets miR-30a-5p/RAB11A to form a ceRNA axis to regulate the proliferation, invasion and EMT (epithelial-mesenchymal transition) process of prostate cancer PC3 cells *via* the classical signaling pathway Wnt/β-catenin, contributing to the understanding of NORAD regulatory mechanisms in prostate cancer. Liu et al. (35) found that the lncRNA PCAT6 sponge miR-326 functions as a ceRNA, acting on the target gene hnRNPA2B1 to regulate prostate cancer proliferation, invasion and neuroendocrine differentiation. Jiang et al. (36) found that lncRNA NEAT1 sponges miR-34a-5p and miR-204a-5p, two miRNAs that act on docetaxel-resistant prostate cancer cells by regulating the target protein ACSL4 *via* a post-translational pathway. This shows promising implications for docetaxel-resistant prostate cancer patients. Li et al. (37) found that the potential transcription factor CDC5L, whose transcriptional activity is dependent on lncRNA NEAT1, was explored using the TCGA database, and CDC5L was used to find the target AGRN, which is also a binding partner of TGFβ1, constituting a transcriptional regulatory circuit of NEAT 1-CDC 5L-AGRN that affects DNA damage and cell cycle and proliferation *via* the TGF-β signaling pathway, adding strong evidence for the mechanism of action of lncRNA NEAT1 in prostate cancer. Liu et al. (38) found that using METTL3 to mediate the m6A methylation modification of lncRNA SNHG7 enhances lncRNA SNHG7 stability, and the stable lncRNA SNHG7 regulates c-Myc expression by recruiting SRSF1, forming a SNHG7/SRSF1/c-Myc axis to regulate glycolysis in prostate cancer cells, providing a new idea for targeted therapy of prostate cancer. Han et al. (39) found that the lncRNA SNHG7 sponge action binds miR-324-3p and regulates WNT2B through sponge effect, constituting a SNHG7/miR-324-3p/WNT2B axis to regulate epithelial-mesenchymal transition in prostate cancer cells, providing a new strategy for the treatment of prostate cancer. Li et al. (40) found that the lncRNA SNHG3 endogenously sponge, miR-577, regulates the target gene SMURF1, forming a SNHG3/miR-577/SMURF1 axis affecting cell viability, proliferation, migration, invasion, EMT processes and apoptosis in prostate cancer cells, providing a new insight into biomarkers of prostate cancer. Wang et al. (41) found that the SNHG3/miR-152-3p/SLC7A11ceRNA axis is closely related to the methionine dependence of prostate cancer cells, and the SNHG3 molecule sponge miR-152-3p, which affects the expression of SLC7A11, thus regulates the methionine dependence of prostate cancer cells to promote the development of prostate cancer, providing a new idea for the role of SNHG3 in prostate cancer.

Although this meta-analysis has a detailed article search strategy and strict inclusion and exclusion criteria, it still has the following shortcomings: (1) The size of samples tested in this study is relatively small, and the sampling error is large, thus, the results are not so stable and reliable, and may lack a certain degree of rigor. (2) Although the same qRT-PCR detection method was used in the included studies, different PCR primer sets were used, which would inevitably lead to the existence of heterogeneity between studies. (3) There is no consensus on the cut-off value of high and low expression of lncRNA among studies, and there were differences in each study, which may affect the relevant results, plus not all articles reported these data. (4) The result of HR and 95% CI were extracted indirectly from the survival curve using the Engauge Digitizer 11.1 software, resulting in a certain amount of inaccuracy. This may increase potential bias. (5) Since the articles did not provide an analysis of the correlation between clinical characteristics and lncRNAs, it was impossible to these analyses. This limits the clinical practical value of lncRNAs to a certain extent. (6) The included studies are all from China, indicating that our findings are not representative of western countries to a certain extent, which limits the study results by region and ethnicity, and leads to potential publication bias. Therefore, well-designed and multi-ethnic clinical studies with larger sample sizes should be carried out in the future.

## Conclusion

In conclusion, this study suggests that lncRNA with pro-cancer effects in prostate cancer cells are significantly associated with lower OS and lower BMSF in prostate cancer bone metastasis. LncRNAs may serve as promising predictors for poor prognosis and clinicopathological features of prostate cancer bone metastasis. However, well-designed and high-quality studies covering different ethnicities with larger sample sizes are still needed to validate these conclusions.

## Data availability statement

The datasets presented in this study can be found in online repositories. The names of the repository/repositories and accession number(s) can be found in the article/supplementary material.

## Author contributions

SS, YZ and SL were responsible for study conception and design. Data collection and statistical analysis were performed by SS, YZ, XZ and SC. The first draft of the manuscript was written by SS and YZ. SC, XZ and SL are responsible for revising the manuscript and all authors contributed to the article and approved the submitted version.
